# Waste water effluent contributes to the dissemination of CTX-M-15 in the natural environment

**DOI:** 10.1093/jac/dku079

**Published:** 2014-05-05

**Authors:** G. C. A. Amos, P. M. Hawkey, W. H. Gaze, E. M. Wellington

**Affiliations:** 1 School of Life Sciences, University of Warwick, Coventry CV4 7AL, UK; 2 Health Protection Agency, West Midlands Public Health Laboratory, Heart of England NHS Foundation Trust, Bordesley Green East, Birmingham, UK; 3 Institute of Microbiology and Infection, Biosciences, University of Birmingham, Birmingham, UK

**Keywords:** antibiotic resistance, β-lactamases, CTX-M, environmental pathogens, carbapenem resistance

## Abstract

**Objectives:**

Multidrug-resistant Enterobacteriaceae pose a significant threat to public health. We aimed to study the impact of sewage treatment effluent on antibiotic resistance reservoirs in a river.

**Methods:**

River sediment samples were taken from downstream and upstream of a waste water treatment plant (WWTP) in 2009 and 2011. Third-generation cephalosporin (3GC)-resistant Enterobacteriaceae were enumerated. PCR-based techniques were used to elucidate mechanisms of resistance, with a new two-step PCR-based assay developed to investigate *bla*_CTX-M-15_ mobilization. Conjugation experiments and incompatibility replicon typing were used to investigate plasmid ecology.

**Results:**

We report the first examples of *bla*_CTX-M-15_ in UK river sediment; the prevalence of *bla*_CTX-M-15_ was dramatically increased downstream of the WWTP. Ten novel genetic contexts for this gene were identified, carried in pathogens such as *Escherichia coli* ST131 as well as indigenous aquatic bacteria such as *Aeromonas media.* The *bla*_CTX-M-15 ­_gene was readily transferable to other Gram-negative bacteria. We also report the first finding of an imipenem-resistant *E. coli* in a UK river.

**Conclusions:**

The high diversity and host range of novel genetic contexts proves that evolution of novel combinations of resistance genes is occurring at high frequency and has to date been significantly underestimated. We have identified a worrying reservoir of highly resistant enteric bacteria in the environment that poses a threat to human and animal health.

## Introduction

Growing evidence suggests anthropogenic activities such as agriculture contribute to environmental reservoirs of resistant bacteria that can directly or indirectly transfer to humans.^1,2^ Waste water treatment plants (WWTPs) process waste from several sources, including human, animal and industrial waste, providing a hotspot for horizontal gene transfer to occur between bacteria from many origins. Few studies have demonstrated the impacts of liquid WWTP effluent on antibiotic resistance loads in rivers, particularly with reference to third-generation cephalosporin (3GC) resistance.^3^ The most common mechanism conferring resistance to 3GCs is the production of plasmid-mediated extended-spectrum β-lactamases (ESBLs), of which the most prevalent are the CTX-M enzymes encoded by *bla*_CTX-M_.^4^ Evidence suggests insertion sequence elements IS*Ecp1* and IS*26* mobilized progenitors of *bla*_CTX-M_ onto plasmids from the chromosome of *Kluyvera* species, a common rhizosphere organism.^5^ Subsequently, plasmid-borne *bla*_CTX-M_ genes have disseminated throughout the Enterobacteriaceae and Gammaproteobacteria.^6^ Currently, there are >145 different genotypes of *bla*_CTX-M_ (http://www.lahey.org/studies), which are often region specific, with *bla*_CTX-M-15_ being the most prevalent in humans worldwide and in the UK.^4^ Rivers are routinely used for the release of WWTP effluent and are a repository for sewage through storm drain overflow. A surveillance study found *bla*_CTX-M-14_ in UK rivers; however, to date, the most clinically important ESBL *bla*_CTX-M-15_ has not been found.^7^ Environmental reservoirs of antibiotic-resistant bacteria are likely to represent a significant exposure risk to humans, through direct contact or indirectly through contaminated drinking water or irrigation of crops. We hypothesize that waste water disposal methods are a contributing factor in resistance gene dissemination in rivers. The current study involves a comparative analysis of 3GC-resistant Enterobacteriaceae upstream and downstream of a WWTP effluent point.

## Materials and methods

### Sampling

Sampling took place in December 2009 and January 2011. Sediment core samples were taken from a river in the UK midlands at three sites in triplicate, 300, 600 and 900 m upstream of a WWTP and three sites in triplicate, 300, 600 and 900 m downstream of a WWTP. The treatment plant served ∼500 000 people and processed >120 million litres of raw sewage per day using a primary settlement tank, secondary activated sludge treatment and final tertiary filtration. Upstream of the treatment plant, geospatial analyses had indicated no other WWTP for ≥10 km. All samples were immediately stored at 4°C and processed within 24 h.

### Viable counts

Sediment from one of each downstream sample site (300, 600 and 900 m) was pooled in equal parts (1 g total) and resuspended in 1 mL of PBS buffer. This was repeated for upstream sediment samples. In total, each of the triplicate sediment samples was pooled to form three downstream samples and three upstream samples. Chromocult Coliform Agar (Merck) was prepared in accordance with the manufacturer's instructions and amended with cefotaxime (2 mg/L) or ceftazidime (16 mg/L). Downstream and upstream samples were plated (200 μL) in triplicate for each antibiotic and unamended Chromocult, before incubating for 24 h at 30°C. Viable plate counts were taken; blue colonies indicated presumptive *Escherichia coli* and pink colonies indicated other coliforms termed presumptive coliforms excluding *E. coli* (PCEs). Reference strains of *E. coli*, *Klebsiella oxytoca*, *Citrobacter freundii*, *Pseudomonas fluorescens* and *Aeromonas media* were used to evaluate the performance of Chromocult at 30°C.

### Bacterial isolation

PCEs and *E. coli* were picked and streaked to obtain pure cultures. The number of isolates obtained for each site differed due to different resistance gene prevalences between sample sites.

### Antimicrobial susceptibility determination

MICs of cefotaxime (1–2048 mg/L) and imipenem (1–32 mg/L) were determined using a broth microdilution method based on CLSI and EUCAST standards as previously described.^8^

### DNA extractions

Isolates were incubated overnight at 30°C in Luria broth (LB) and DNA was extracted using a Nucleospin Blood Kit (Macherey-Nagel) in accordance with the manufacturer's instructions.

### Identification of bacteria

Bacteria were first identified by sequencing PCR products obtained using the universal 27F and 1525R 16S rRNA primers.^9^ Further identification of Enterobacteriaceae was performed by partial sequencing of *dnaJ* as previously described.^10^ *Aeromonas* spp*.* were identified using partial sequencing of *gyrB*.^11^ *E. coli* strains were typed using the Achtman scheme.^12^

### Detection of 3GC resistance genes in isolates

PCR amplification of *bla* genes, including *bla*_TEM_, *bla*_SHV_ and *bla*_CTX-M_, was performed as previously described.^13^

### Analysis of *bla*_CTX-M-15_ flanking regions

Characterization of the regions flanking *bla*_CTX-M_ was performed with PCR as previously described.^14^

Further analysis of flanking regions unidentified by conventional PCR was done by modifying the two-step gene-walking method (please see the Supplementary data at *JAC* Online)^15^ and newly designed primers (CTXD-F, 5′-TCACCCAGCCTCAACCTAAG-3′; and CTXD-R, 5′-CGCTCATCAGCACGATAAAG-3′) were used to detect duplications (please see the Supplementary data at *JAC* Online).

### Conjugation assays

*E. coli* DH10B (Str^R^) with induced rifampicin resistance was used as a recipient strain for solid conjugal mating assays with positive *bla*_CTX-M-15_ strains as donors. Transconjugants were selected using LB plates amended with streptomycin (100 mg/L), rifampicin (100 mg/L) and cefotaxime (2 mg/L). Positive transconjugants were confirmed using the PCR primer pair CTX-F and CTX-R.

### Plasmid replicon typing

Plasmid replicon types in *bla*_CTX-M-15_-positive strains were identified using a PCR-based method.^16^ Strains with identical replicon types were further analysed using restriction fragment length polymorphism (RFLP) (please see the Supplementary data at *JAC* Online).

### Statistical analysis

All statistics were performed using Genstat 15th edition SP1 (VSN International). For comparison of means, log counts were checked for normal distribution using the Shapiro–Wilk test followed by analysis using a paired-sample *t*-test. Proportions were compared using Fisher's exact test.

## Results

### Viable plate counts

**Figure 1.**
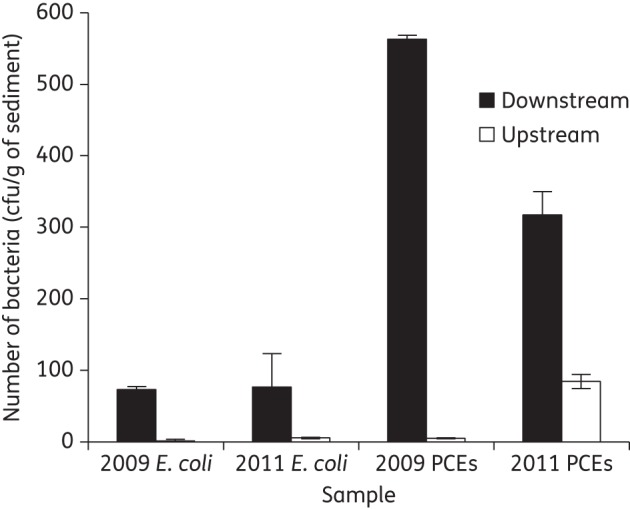
Counts of 3GC-resistant presumptive *E. coli* and PCEs from samples collected downstream and upstream of a WWTP in 2009 and 2011. Error bars are ± standard errors of biological replicates.

There was a significant increase in the numbers of 3GC-resistant presumptive *E. coli* and PCEs in the river sediment downstream of effluent discharge in both 2009 and 2011(Figure 1) (*t*-test *P* < 0.0001 in all cases). No significant difference was recorded in numbers of 3GC-resistant *E. coli* in downstream samples between 2009 and 2011; however, there was a significant increase in numbers of 3GC-resistant PCEs in upstream samples between 2009 and 2011 (*t*-test *P* < 0.0001 in all cases). The mean average number of total coliforms in 2011 was 4 × 10^5^/g of wet sediment downstream and 2 × 10^5^/g of wet sediment upstream. The mean average number of *E. coli* in 2011 was 8 × 10^3^/g of wet sediment downstream and 4 × 10^3^/g of wet sediment upstream. From this, we can calculate that 0.95% of *E. coli* were resistant to 3GCs downstream compared with 0.13% of *E. coli* upstream. Similarly, for coliforms there were 0.079% resistant downstream compared with 0.042% upstream.

### Identification of bacteria

All isolated 3GC-resistant presumptive *E. coli* (*n* = 41) were confirmed as *E. coli* by sequencing *dnaJ* PCR products (Table 1). Isolated 3GC-resistant PCE isolates (*n* = 19) were also identified using *dnaJ* (Table 1). In both downstream and upstream samples, a proportion of PCEs (18.2% downstream and 50% upstream) were identified as members of the *Aeromonas* genus, species *A. media*. Multilocus sequence typing (MLST) analysis of *E. coli* isolates revealed uncharacterized sequence types (STs) particularly from upstream samples (80%), indicating the existence in this environment of novel STs. Downstream of the WWTP, the human-associated ST3103 and ST38 were codominant in 2009, but neither of these STs was detected in 2011 samples, which were dominated by the well-recognized human disease-associated types ST131 (20%) and ST167 (25%) [Table 2 and Table S1 (available as Supplementary data at *JAC* Online)].

**Table 1.** Prevalence of different β-lactamases determined by PCR screening

Sample site	Organism	Number isolated	*bla*_CTX-M_ prevalence (%)	*bla*_TEM_ prevalence (%)	*bla*_SHV_ prevalence (%)
Downstream 2009	*E. coli*	11	100	100	0
Downstream 2011	*E. coli*	20	100	100	0
Downstream 2011	*K. oxytoca*	3	100	100	0
Downstream 2011	*C. freundii*	3	100	100	0
Downstream 2011	*C. braakii*	1	100	100	0
Downstream 2011	*Raoultella ornithinolytica*	1	0	100	0
Downstream 2011	*A. media*	2	0	0	0
Downstream 2011	*P. fluorescens*	1	100	100	0
Upstream 2011	*E. coli*	10	100	100	0
Upstream 2011	*K. oxytoca*	1	0	0	0
Upstream 2011	*C. freundii*	3	33.3	66.6	33.3
Upstream 2011	*A. media*	4	50	100	0

**Table 2.** Molecular characterization of 52 *bla*_CTX-M_-positive isolates

CTX-M genotype (genetic context group)	Composition of isolates in each genetic context	Associated plasmid Inc replicon types	Cefotaxime MIC (mg/L)	Transfer through conjugation
2009 isolates				
CTX-M-1	downstream: *E. coli* ST38 (5)	FIB	1024–2048	yes
CTX-M-15 international environment (Group A)	downstream: *E. coli* ST3103 (4)	F, K, IL/IY	>2048	yes
CTX-M-15 Group I	downstream: *E. coli* ST New (1)	FIB, I1/IY	64	yes
CTX-M-15 Group J	downstream: *E. coli* ST3103 (1)	FIB, I1/IY	>2048	yes
2011 isolates				
CTX-M-15 international environment (Group A)	downstream: *C. braakii* (1), *C. freundii* (1), *E. coli* incl. ST131 (3), ST167 (1) and ST New (2)	FIB, K, HI2, A/C, FIIA	16–2048	yes
	upstream: *E. coli* incl. ST131 (1) and ST New (3)			
CTX-M-15 Group I	downstream: *K. oxytoca* (2)	FIIA, HI2	>2048	yes
CTX-M-15 Group K	downstream: *C. freundii* (2), *E. coli* incl. ST1060 (1) and ST167 (1)	FIA, FIB, K, IL/IY, A/C	1024–2048	yes
	upstream: *A. media* (1) and *E. coli* ST New (1)			
CTX-M-15 Group L	upstream: *C. freundii* (1)	FIA	64	yes
CTX-M-15 Group M	downstream: *E. coli* ST New (1)	HI2	16	yes
CTX-M-15 Group N	downstream: *E. coli* ST167 (3) and *K. oxytoca* (1)	FIB, K	1024–2048	yes
	upstream: *A. media* (1)			
CTX-M-15 Group O	downstream: *E. coli* ST1421 (1)	FIB, IL/IY	>2048	yes
CTX-M-15 Group P	downstream: *E. coli* ST New (1)	F, K	128	yes
CTX-M-15 Group Q	downstream: *E. coli* incl. ST131 (1) and ST New (2)	FIB, FIIA, HI2, K	>2048	yes
CTX-M-15 Group R	upstream: *E. coli* ST New (1)	FIB, HI2	16	no
CTX-M-15 unidentified groups	downstream: *E. coli* ST New (3) and *P. fluorescens* (1)	F, FIA, FIIA, FIB, K	32–512	yes
	upstream: *E. coli* incl. ST410 (1) and ST New (3)			

Downstream: isolates recovered downstream of WWTP. Upstream: isolates found upstream of WWTP. ST is the result from MLST with New referring to an isolate with no MLST type matching the MLST database. GenBank accession numbers: Group I, KF155153; Group J, KF155154; Group K, KF155155; Group L, KF155156; Group M, KF155157; Group N, KF155158; Group O, KF155159; Group P, KF155160; and Group Q, KF155161.

### Detection and characterization of β-lactamases in resistant isolates

All *E. coli* were positive for *bla*_CTX-M_ and *bla*_TEM_, but negative for *bla*_SHV_ (Table 1). Sequencing revealed all *bla*_CTX-__M_-bearing isolates in 2011 carried *bla*_CTX-M-15_ and 54.5% of isolates in 2009 carried *bla*_CTX-M-15_ with the remainder carrying *bla*_CTX-M-1_.

### Analysis of genetic variation in bla_CTX-M_ flanking regions

**Figure 2.**
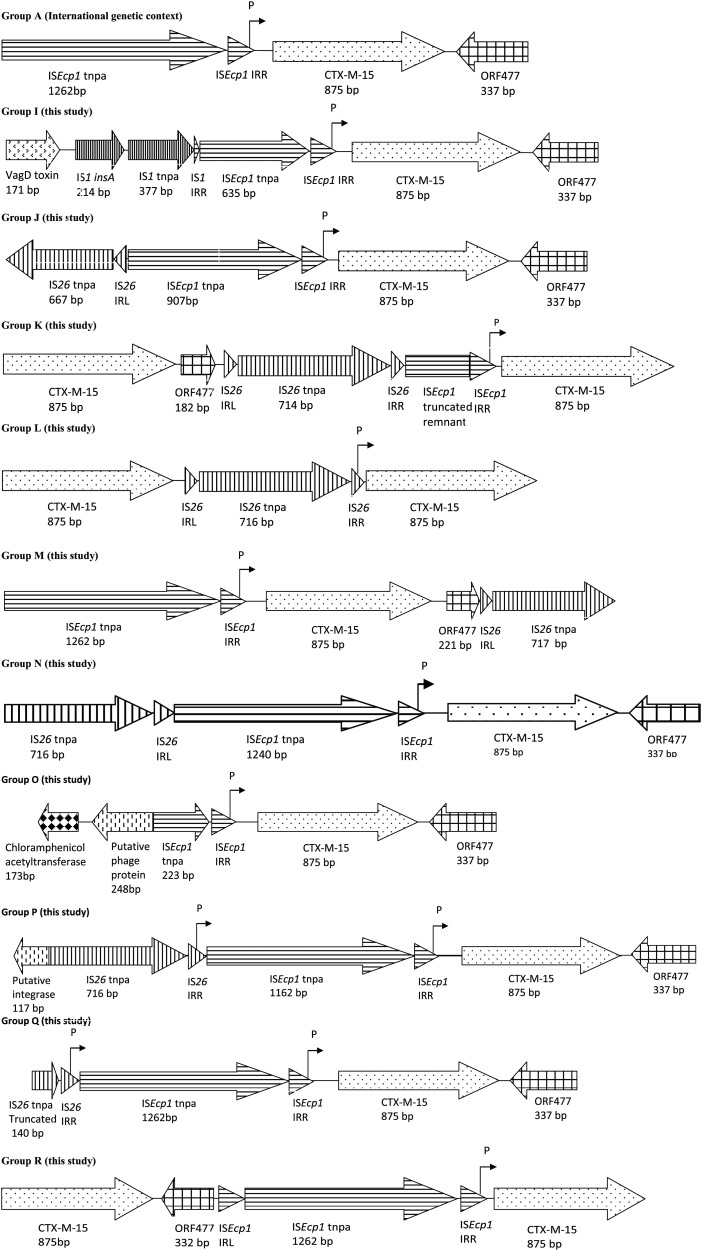
Flanking regions of *bla*_CTX-M-15_ recovered in isolates obtained during this study as confirmed by two-step PCR and sequencing. Nomenclature is an extension of a previously defined typing system.^14^

A total of 11 different genetic contexts were found in association with *bla*_CTX-M-15_ (Figure 2, Table 2 and Table S1), 10 of which were novel and denoted I–R in keeping with the nomenclature as previously described.^14^ A total of five genetic contexts were found upstream of the WWTP and nine genetic contexts were found downstream of the WWTP. Three of the genetic contexts were downstream and upstream of the WWTP simultaneously: Group A was the most prevalent and accounted for 67% of the total context type in 2009 and 29% in 2011; Group N was found in *A. media* in upstream samples, but in *E. coli* and *K. oxytoca* in downstream samples; and Group K was found in *A. media* and *E. coli* in upstream samples, but in *E. coli* and *C. freundii* in downstream samples. Three groups carried multiple copies of *bla*_CTX-M-15_: Group K was the only group recovered downstream of the WWTP that consisted of a repeated *bla*_CTX-M-15_; and the other two contexts (Groups L and R) both came from upstream of the WWTP. The CTXD-F and CTXD-R primers allowed for detection of *bla*_CTX-M-15_ repeats found in Groups K, L and R; however, several groups were unresolved even after two-step gene walking, due to multiple copies of *bla*_CTX-M-15_ and repeat regions. Aside from Group A, Group I was the only other group recorded in both 2009 and 2011, but in *E. coli* in 2009 and *K. oxytoca* in 2011.

Several of the groups contained new elements not previously associated with *bla*_CTX-M-15_ flanking regions, such as IS*1*, putative phage proteins, toxin genes and resistance genes to other antibiotics (Figure 2).

### Relationship between genetic context and MIC

Isolates containing seven of the new genetic contexts had MICs of cefotaxime >1024 mg/L. Significantly higher MICs were characteristic of isolates found in downstream samples compared with upstream samples (*t*-test *P* = 0.024); however, promoter analysis (Figure S1, available as Supplementary data at *JAC* Online) revealed that all but one of the contexts shared the same promoter region of *bla*_CTX-M-15_ as Group A. One *E. coli* strain had a high level of resistance to imipenem (>32 mg/L), though this was not conferred through *bla*_CTX-M-15_ (Table S1).

### Plasmid diversity and relationships to genetic contexts

**Figure 3.**
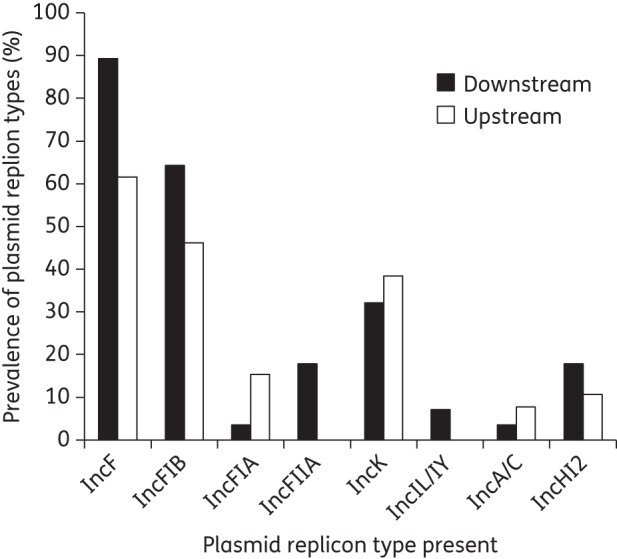
Prevalence of different plasmid replicon types as validated by PCR for 41 *bla*_CTX-M-15_-bearing isolates from river sediment downstream of WWTP effluent and upstream of WWTP effluent in 2011.

In both years, a number of different replicon types were associated with CTX-M-carrying strains (Figure 3, Table 2 and Table S1). IncF was the most prevalent replicon type both downstream and upstream of the WWTP. The same genetic context was regularly recorded in strains carrying different replicon types, with the Group A context being associated with the most diverse range of replicons (seven types; Table 2). PFGE analysis was used to compare isolates with the same *bla*_CTX-M-15­_ context and plasmid replicons (Table S1). Group N was carried by the same plasmid upstream and downstream of the WWTP across different families; initially upstream in *A. media* and downstream in *K. oxytoca* and *E. coli*.

Conjugation experiments revealed all but two of the *bla*_CTX-__M_-bearing isolates could transfer this gene at frequencies between 10^−3^ and 10^−7^, with expression of cefotaxime resistance ≥2 mg/L in transconjugants. Conjugation rates varied depending on the host background and the plasmid composition (Table S1).

## Discussion

Tertiary treatment by WWTPs is the most rigorous level of waste water treatment in the UK as set out by the Water Services Regulation Authority (Ofwat).^17^ We demonstrated that even with this level of treatment, WWTP effluent has a significant impact on numbers of 3GC-resistant bacteria in river sediment communities. As well as an increase in the total numbers of 3GC-resistant bacteria, the WWTP had an impact on the prevalence of 3GC resistance in bacteria with a 7-fold increase in the prevalence of 3GC-resistant *E. coli*. The cause of 3GC resistance predominantly resulted from the dissemination of *bla*_CTX-M-15_. This is the first report of *bla*_CTX-M-15_ in UK river waters and represents a worrying trend as this gene is the most common ESBL in *E. coli* and *Klebsiella* spp*.* causing clinical disease.^4^ Novel hosts were isolated across the Gammaproteobacteria, including *Citrobacter braakii*, *A. media* and *P. fluorescens*, none of which has previously been reported as a carrier of *bla*_CTX-M-15_. Many of the resistant bacteria were pathogens, such as *E. coli* ST131, ST167 and ST38, *C. freundii* and *K. oxytoca*. In particular, the finding of *bla*_CTX-M-15_ in the pandemic pathogen *E. coli* ST131 as a viable and significant reservoir in environmental samples represents a serious threat to human health. This supports recent findings of the threat that rivers pose to human health highlighted by a study in which one-third of people swimming in areas of the River Thames suffered gastrointestinal illness.^18^ In addition, we report the first finding of an imipenem-resistant *E. coli* in a UK river, an indication of the emerging spread of carbapenem resistance in the environment, which is a great cause for concern.

We demonstrated that there was high genetic diversity in *bla*_CTX-M-15_ carriage and hypothesize that such an unprecedented diversity can be attributed to the direct introduction of bacteria by WWTP effluent possibly combined with *in situ* selection either in the river or WWTP. Selection is likely to be aided by antibiotic and detergent residues that have previously been detected in WWTP effluent as well as the high density of bacteria present in WWTPs, which will facilitate cell-to-cell contact.^3,19^ This hypothesis is supported by plasmid analyses as replicon typing revealed eight types present in isolates carrying *bla*_CTX-M-15_. Of particular concern is the frequency (46%) at which multiple plasmid replicons were colocalized in one isolate. This would allow for interplasmid transfer of *bla*_CTX-M-15_ through transposition and homologous recombination and each different genetic context of *bla*_CTX-M-15_ may be indicative of a transfer event.^20^

The carriage of multiple plasmid incompatibility groups will contribute to higher conjugation rates. This resulted in conjugation frequencies that were higher than reported for similar studies conducted with clinical strains and plasmids.^21^ The extensive mobility of plasmids was further emphasized by the recovery of identical plasmids in diverse backgrounds. Of concern was the pool of plasmids shared between hosts regarded as clinical bacteria and those regarded as indigenous to the river environment.

We have demonstrated repeated evidence of the significant introduction of clinically relevant ESBL-producing bacteria by WWTP effluent into a UK river. Many of the pathogens had novel *bla*­_CTX-M-15_ flanking regions, including *E. coli* ST131-carrying Group Q. The prevalence of human-associated bacteria with a high diversity of *bla*_CTX-M-15_ flanking regions downstream of the WWTP supports the hypothesis that community carriage is more extensive than currently thought.^22^ The change in prevalence of *E. coli* STs between 2009 and 2011, with ST131 becoming one of the most dominant STs, is likely a reflection of the clonal spread of this ST in the human population.^22^

An increase in the number of 3GC-resistant coliforms upstream between 2009 and 2011 is potentially a consequence of faecal contamination from surrounding farm environments. This timescale coincides with the detection of the ESBL gene *bla*_CTX-M-1_ in UK cattle, chickens, turkeys and most recently dogs.^23^ The gene *bla*_CTX-M-15­_ has been detected throughout Europe in companion animals and a diverse range of wild birds.^24^ Whilst it is not possible to determine the direction of spread from humans to animals, the significant environmental reservoir in rivers will impact both. In conclusion, we report a reservoir of *bla*_CTX-M-15_ in a UK river with clear evidence of extensive recombination of the gene within plasmid populations. A growing environmental reservoir presents a risk to human health, with evidence implicating WWTP effluent as a major contributor to the formation of this reservoir.

Further research is needed into sewage treatment systems that result in minimal introduction of resistant bacteria and selecting agents such as antibiotic residues and quaternary ammonium compounds. Stricter regulations and higher levels of treatment are needed if we are to halt the rise of antibiotic resistance in the environment.

## Funding

This work was supported by the Natural Environment Research Council (grant NE/E004482/1). G. C. A. A. was supported by a BBSRC studentship. W. H. G. has been supported by the ERDF and ESF since moving to the University of Exeter.

## Transparency declarations

None to declare.

### Author contributions

G. C. A. A., W. H. G. and E. M. W. designed the research, G. C. A. A. performed the research, all authors analysed the data and all authors contributed to writing the paper.

## Supplementary data

Supplementary methods, Table S1 and Figure S1 are available as Supplementary data at *JAC* Online (http://jac.oxfordjournals.org/).

Supplementary Data
