# Supplementary material for: Waste water effluent contributes to the dissemination of CTX-M-15 in the natural environment
Source: J Antimicrob Chemother. 2014 May 5;69(7):1785–91. doi: 10.1093/jac/dku079 (PMC4054988; doi:10.1093/jac/dku079)
Supplement: Supplementary Data [file supp_69_7_1785__index.html]

Waste water effluent contributes to the dissemination of CTX-M-15 in the natural environment — Waste water effluent contributes to the dissemination of CTX-M-15 in the natural environment — Supplementary Data 

# Waste water effluent contributes to the dissemination of CTX-M-15 in the natural environment

## Supplementary Data

Supplementary Data

**Files in this Data Supplement:**

- Supplementary Data - Docx file
