# Supplementary material for: Waste water effluent contributes to the dissemination of CTX-M-15 in the natural environment
Source: J Antimicrob Chemother. 2014 May 5;69(7):1785–91. doi: 10.1093/jac/dku079 (PMC4054988; doi:10.1093/jac/dku079)
Supplement: Supplementary Data [file supp_dku079_dku079supp.docx]

**Supplementary data**

**Supplementary methods**

***Modified two-step gene walking method***

Modified two-step gene walking method initially uses solely primer CTX-R 5′-CCGTTTCCGCTATTACAAAC-3 in a reaction including 0.25 µL taq, 5 µL reaction buffer, 5 µL dNTPs, 2 µL DNA template, and primer CTX-R at a concentration of 1.0 µΜ. Cycling conditions in stage 1 94ºC 4 min ; in stage 2 20 cycles of 94ºC 30 s, 50ºC 30 s, 72ºC 4 min; in stage 3 1 cycle of 94ºC 30 s, 35ºC 30 s, 72ºC 4 min, in stage 4 20 cycles of 94ºC 30 s, 50ºC 30 s, 72ºC 4 min, in stage 5 72ºC for 10 min. Resulting PCR products were fractionated using 0.8 % agarose gel with electrophoresis conditions of 80 v for 3 hours in TAE buffer. Clean bands above 1.5 kb in size were cut and purified using a gel extraction kit (Qiagen). Resulting bands were either subcloned into pGEM-T (promega) and sequenced using primer CTXD-R 5’- CGCTCATCAGCACGATAAAG – 3’(this study), or directly sequenced using CTXD-R 5’- CGCTCATCAGCACGATAAAG – 3’. Resulting sequences were used for subsequent primer walking.

***Detection of bla_CTX-M-15_ duplications***

Newly designed primers CTXD-F 5’–TCACCCAGCCTCAACCTAAG-3’and CTXD-R 5’- CGCTCATCAGCACGATAAAG – 3’ were used to detect duplications of *bla*_CTX-M-15_. PCR reactions consisted of a 50 µL reaction using Takara Ex Taq (Clontech) reagents including 0.25 µL taq, 5 µL reaction buffer, 5 µl dNTPs, 2 µL DNA template, and primers at a concentration of 0.8 µΜ. PCR cycle conditions in stage 1 95ºC 2 min; in stage 2 40 cycles of 95ºC 30 s, 60ºC 30 s, 72ºC 6 min; stage 3 72ºC for 10 min.

***RFLP for analysis of plasmid similarity***

Strains with identical replicon types were further analysed using RFLP; plasmid extraction was performed using a modified protocol for the Qiagen plasmid Midi kit (Qiagen), DNA (3 µg) was digested with 15 units HindIII-HF (New England Biolabs) for 4 h, digested plasmids were analysed by PFGE using a 1.0% agarose gel (pulsed-field grade; Bio-Rad) in 0.5 x TBE buffer, with electrophoresis performed using a CHEF mapper (Biorad). Conditions used were 6.0 V cm^-1^ for 20.18 h at 14 ºC with an initial switch time of 0.47 s and a finishing switch time of 17.33 s.

**Table S1.** Molecular characterization of 52 *bla*_CTX-M_ positive isolates

| **Isolate number, CTX-M genotype (genetic context group)** | **Species** | ***E. coli* MLST** | **Plasmid inc rep types** | **PFGE profiling of identical rep types** | **MIC (mg / L)** | | **Conjugation Frequencies** |
| --- | --- | --- | --- | --- | --- | --- | --- |
|  |  |  |  |  | **Cefotaxime** | **Imipenem** |  |
| **2009 Isolates** |  |  |  |  |  |  |  |
| **CTX-M-15 International environment (Group A)** |  |  |  |  |  |  |  |
| 1 | *E. coli* | ST3103 | F |  | >2048 | <1 | 4.33 x 10-4 |
| 2 | *E. coli* | ST3103 | F, K, I1/IY |  | >2048 | <1 | 1.8 x 10-5 |
| 3 | *E. coli* | ST3103 | FIB, I1/IY |  | >2048 | <1 | 1.2 x 10-4 |
| 4 | *E. coli* | ST3103 | FIB, K, I1/IY |  | >2048 | <1 | 1.66 x 10-4 |
| **CTX-M-15 Group I** |  |  |  |  |  |  |  |
| 5 | *E. coli* | New | FIB, I1/IY |  | 64 | >32 | 2.79 x 10-6 |
| **CTX-M-1** |  |  |  |  |  |  |  |
| 6 | *E. coli* | ST38 | FIB | Unique | 1024 | <1 | 4.8 x 10-5 |
| 7 | *E. coli* | ST38 | FIB | Identical to 9 | >2048 | <1 | 2.7 x 10-5 |
| 8 | *E. coli* | ST38 | FIB | Unique | 1024 | <1 | 1.04 x 10-4 |
| 9 | *E. coli* | ST38 | FIB | Identical to 7 | >2048 | <1 | 2.7 x 10-5 |
| 10 | *E. coli* | ST38 | FIB | Unique | >2048 | <1 | 5.35 x 10-5 |
| **CTX-M-15 Group J** |  |  |  |  |  |  |  |
| 11 | *E. coli* | ST3103 | FIB, I1/IY |  | >2048 | <1 | 1.8 x10-5 |
| **2011 Isolates** |  |  |  |  |  |  |  |
| **CTX-M-15 International environment (Group A)** |  |  |  |  |  |  |  |
| 52 US | *E. coli* | ST131 | FIB | Unique | >2048 | <1 | 1.3 x 10-4 |
| 12 US | *E. coli* | New | FIB, K, HI2 |  | 64 | <1 | 3.3 x 10-7 |
| 13 US | *E. coli* | New | K |  | 16 | <1 | Fail |
| 42 US | *E. coli* | New | HI2, K |  | 1024 | <1 | 4.34 x 10-6 |
| 35 | *C. braakii* |  | FIB |  | 1>2048 | <1 | 7.10 x 10-4 |
| 14 | *C. freundii* |  | A/C, K |  | >2048 | 2 | 2.91 x 10-5 |
| 15 | *E. coli* | ST131 | FIB | Identical to 16 | 128 | <1 | 3.5 x 10-5 |
| 16 | *E. coli* | ST131 | FIB | Identical to 15 | 256 | <1 | 4.6 x 10-5 |
| 17 | *E. coli* | New | FIB, HI2 |  | 16 | <1 | 6 x 10-4 |
| 18 | *E. coli* | New | FIB | Unique | 16 | <1 | 8.2 x 10-7 |
| 19 | *E. coli* | ST167 | FIIA |  | 1024 | <1 | 2.42 x 10-4 |
| 20 | *E. coli* | ST131 | FIB | Unique | >2048 | <1 | 2.63 x 10-5 |
| **CTX-M-15 Group I** |  |  |  |  |  |  |  |
| 37 | *K. oxytoca* |  | FIIA, HI2 | Unique | >2048 | <1 | 3 x 10-3 |
| 38 | *K. oxytoca* |  | FIIA, HI2 | Unique | >2048 | <1 | 4.4 x 10-4 |
| **CTX-M-15 Group K** |  |  |  |  |  |  |  |
| 21 | *E. coli* | ST1060 | FIA |  | 1024 | <1 | 6.25 x 10-4 |
| 22 | *C. freundii* |  | FIB, K | Identical to 16 | >2048 | <1 | 9.4 x 10-5 |
| 23 | *C. freundii* |  | FIB , I1/IY |  | >2048 | <1 | 2.85 x 10-5 |
| 24 US | *E. coli* | New | FIB |  | 1024 | <1 | 1.7 x 10-7 |
| 25 US | *A. media* |  | A/C |  | 1024 | <1 | 1 x 10-5 |
| 26 | *E. coli* | ST167 | FIB, K | Identical to 12 | 1024 | <1 | 4.39 x 10-4 |
| **CTX-M-15 Group L** |  |  |  |  |  |  |  |
| 27 US | *C. freundii* |  | FIA |  | 64 | <1 | 3.44 x 10-6 |
| **CTX-M-15 Group M** |  |  |  |  |  |  |  |
| 28 | *E. coli* | New | HI2 |  | 16 | <1 | 6.7 x 10-6 |
| **CTX-M-15 Group N** |  |  |  |  |  |  |  |
| 29 US | *A. media* |  | FIB, K | Identical to 30,31,32,33 | >2048 | <1 | 3.2 x 19-5 |
| 30 | *K. oxytoca* |  | FIB, K | Identical to 29,31,32, 23 | 1024 | <1 | 1 x 10-4 |
| 31 | *E. coli* | ST167 | FIB, K | Identical to 29,30,32, 33 | >2048 | <1 | 3 x 10-4 |
| 32 | *E. coli* | ST167 | FIB K | Identical to 29,30,31, 33 | >2048 | <1 | 4 x 10-4 |
| 33 | *E. coli* | ST167 | FIB K | Identical to 29,30,31,32 | >2048 | <1 | 3 x 10-4 |
| **CTX-M-15 Group O** |  |  |  |  |  |  |  |
| 34 | *E. coli* | ST1421 | FIB, I1/IY |  | >2048 | <1 | 8 x 10-5 |
| **CTX-M-15 Group P** |  |  |  |  |  |  | 1.32 x 10-3 |
| 36 | *E. coli* | New | F, K |  | 128 | <1 |  |
| **CTX-M-15 Group Q** |  |  |  |  |  |  |  |
| 39 | *E. coli* | ST131 | FIB |  | >2048 | <1 | 1.78 x 10-4 |
| 40 | *E. coli* | New | FIIA, HI2 |  | >2048 | <1 | 6 x 10-5 |
| 41 | *E. coli* | New | FIB, K |  | >2048 | <1 | 1.25 x 10-4 |
| **CTX-M-15 Group R** |  |  |  |  |  |  |  |
| 43 US | *E. coli* | New | FIB, HI2 |  | 16 | 2 | Fail |
| **Unidentified Group** |  |  |  |  |  |  |  |
| 44 US | *E. coli* | New | K |  | 128 | <1 | 2 x 10-7 |
| 45 US | *E. coli* | ST410 | Unassigned |  | 128 | <1 | 9.35 x 10-6 |
| 46 US | *E. coli* | New | FIA |  | 32 | <1 | 6.00 x 10-5 |
| 47 US | *E. coli* | New | FIB |  | 512 | <1 | 1.4 x 10-7 |
| 48 | *P. fluorescens* |  | FIIA |  | 512 | <1 | 9 x 10-5 |
| 49 | *E. coli* | New | FIB |  | 32 | <1 | 4 x 10-6 |
| 50 | *E. coli* | New | FIB |  | 32 | <1 | 4 x 10-6 |
| 51 | *E. coli* | New | F |  | 128 | <1 | 3.66 x 10-5 |

Isolate number; US refers to isolates from upstream of the WWTP plant, with all others isolated from downstream of the WWTP. MLST type; New refers to an isolate with no MLST type matching the MLST database. Plasmid inc rep types; Unassigned refers to an isolate with no known inc rep type. Plasmid PFGE profiling performed on isolates with identical CTX-M genetic contexts and plasmid rep types; ‘unique’ indicates isolates had plasmids which did not match those from any other isolate, ‘identical to’ refers to isolates harbouring plasmids with identical PFGE patterns. Genbank accession numbers; Group I KF155153, Group J KF155154, Group K KF155155, Group L KF155156, Group M KF155157, Group N KF155158, Group O KF155159, Group P KF155160, Group Q KF155161.

-35 IS*eCP1* -10 IS*ecp1*


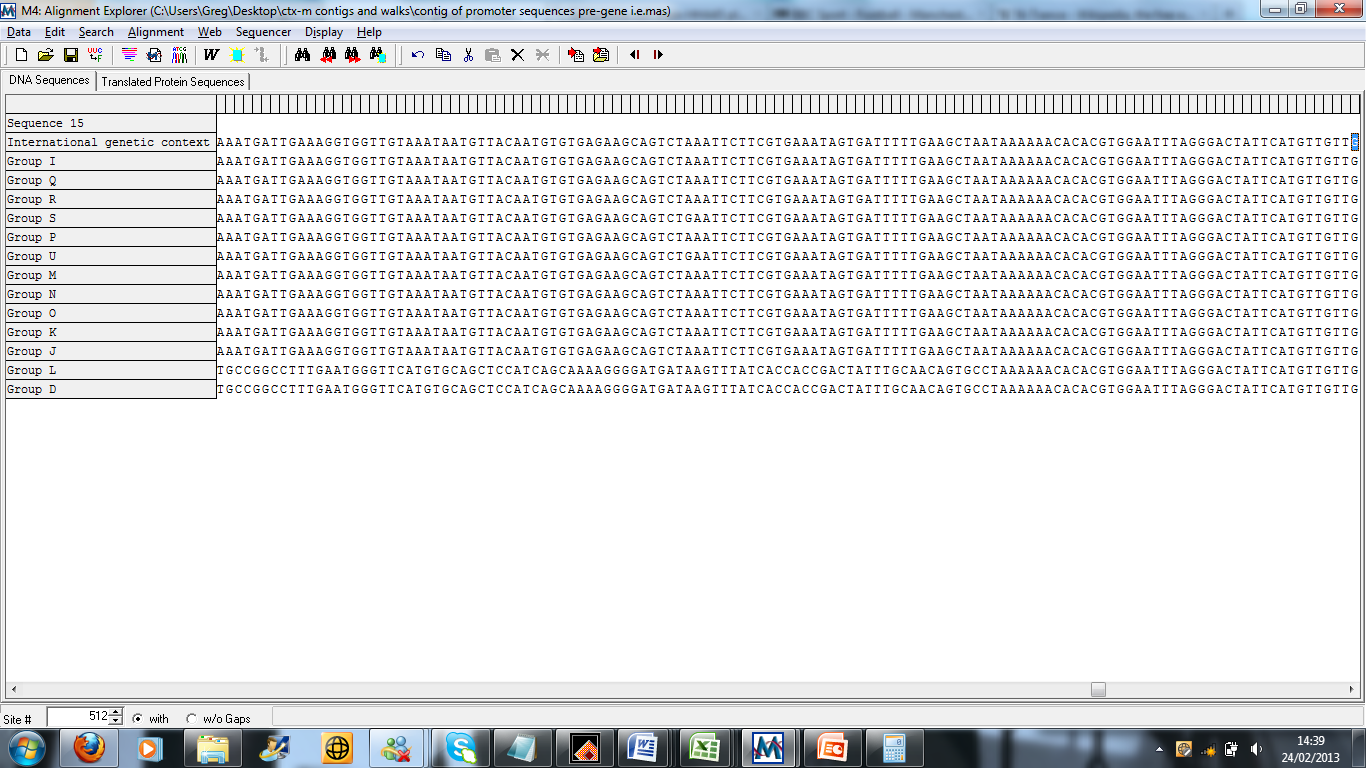


Start of CTX-M-15

- 35 IS*26* -10 IS*26*


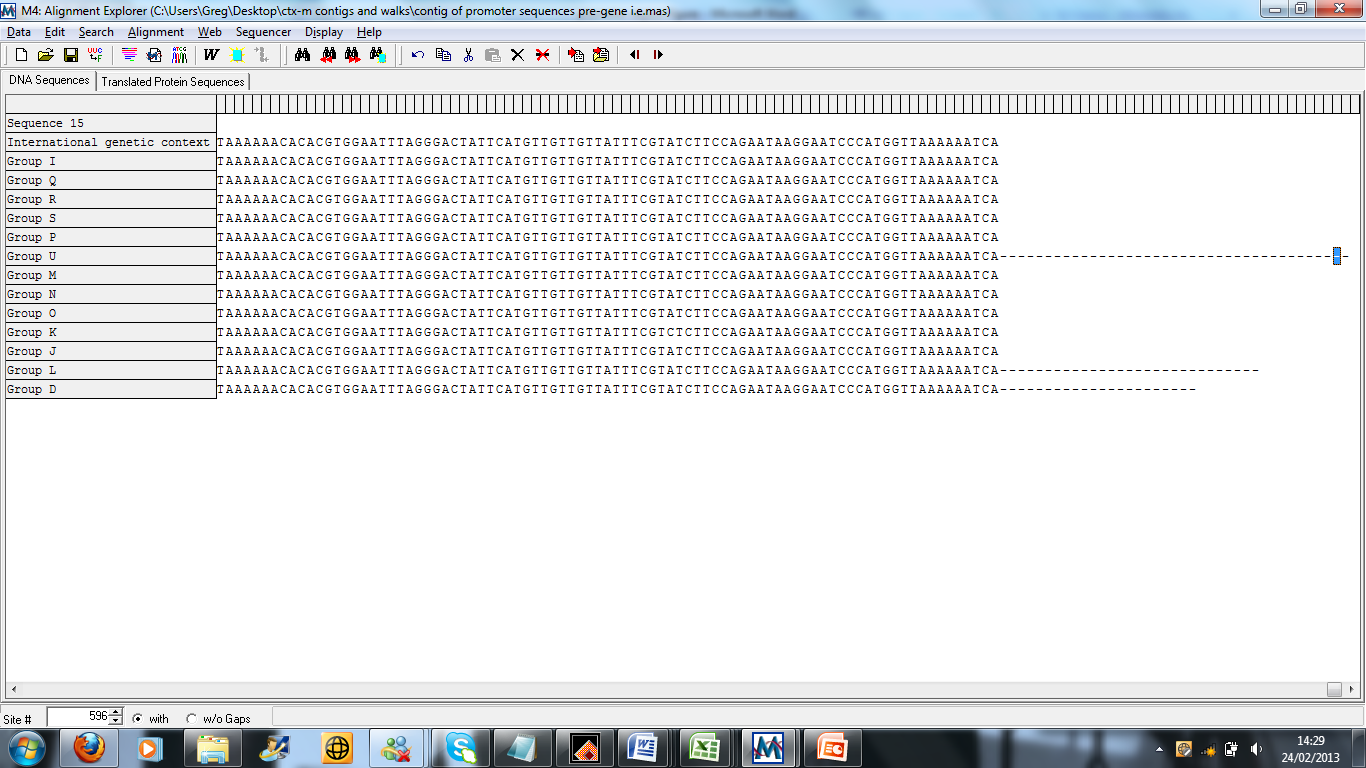


**Figure S1.** Results from promoter analyses of different *bla*_CTX-M-15­_ flanking regions. Conserved regions of genetic contexts which share IR of IS*Ecp1* have the same promoter region as the International genetic context (Group A). Conserved regions of genetic context L has a promoter from the IR of IS*26* and uses the same promoter seen in Group D.^14^
